# The Eukaryotic Host Factor 14-3-3 Inactivates Adenylate Cyclase Toxins of Bordetella bronchiseptica and B. parapertussis, but Not B. pertussis

**DOI:** 10.1128/mBio.00628-18

**Published:** 2018-08-28

**Authors:** Aya Fukui-Miyazaki, Hirono Toshima, Yukihiro Hiramatsu, Keisuke Okada, Keiji Nakamura, Keisuke Ishigaki, Naoaki Shinzawa, Hiroyuki Abe, Yasuhiko Horiguchi

**Affiliations:** a Department of Molecular Bacteriology, Research Institute for Microbial Diseases, Osaka University, Suita, Osaka, Japan; Harvard Medical School

**Keywords:** 14-3-3, *Bordetella*, CyaA

## Abstract

Bordetella pertussis, Bordetella bronchiseptica, and Bordetella parapertussis share highly homologous virulence factors and commonly cause respiratory infections in mammals; however, their host specificities and disease severities differ, and the reasons for this remain largely unknown. Adenylate cyclase toxin (CyaA) is a homologous virulence factor that is thought to play crucial roles in *Bordetella* infections. We herein demonstrate that CyaAs function as virulence factors differently between B. bronchiseptica/B. parapertussis and B. pertussis. *B*. *bronchiseptica* CyaA bound to target cells, and its enzyme domain was translocated into the cytosol similarly to *B*. *pertussis* CyaA. The hemolytic activity of *B*. *bronchiseptica* CyaA on sheep erythrocytes was also preserved. However, in nucleated target cells, *B*. *bronchiseptica* CyaA was phosphorylated at Ser^375^, which constitutes a motif (RSXpSXP [pS is phosphoserine]) recognized by the host factor 14-3-3, resulting in the abrogation of adenylate cyclase activity. Consequently, the cytotoxic effects of *B*. *bronchiseptica* CyaA based on its enzyme activity were markedly attenuated. *B*. *parapertussis* CyaA carries the 14-3-3 motif, indicating that its intracellular enzyme activity is abrogated similarly to *B*. *bronchiseptica* CyaA; however, *B*. *pertussis* CyaA has Phe^375^ instead of Ser, and thus, was not affected by 14-3-3. In addition, *B*. *pertussis* CyaA impaired the barrier function of epithelial cells, whereas *B*. *bronchiseptica* CyaA did not. Rat infection experiments suggested that functional differences in CyaA are related to differences in pathogenicity between B. bronchiseptica/*B*. *parapertussis* and B. pertussis.

## IMPORTANCE

Bordetella pertussis, B. bronchiseptica, and B. parapertussis are bacterial respiratory pathogens that are genetically close to each other and produce many homologous virulence factors; however, their host specificities and disease severities differ, and the reasons for this remain unknown. Previous studies attempted to explain these differences by the distinct virulence factors produced by each *Bordetella* species. In contrast, we indicated functional differences in adenylate cyclase toxin, a homologous virulence factor of *Bordetella*. The toxins of B. bronchiseptica and presumably B. parapertussis were inactivated by the host factor 14-3-3 after phosphorylation in target cells, whereas the B. pertussis toxin was not inactivated because of the lack of the phosphorylation site. This is the first study to show that 14-3-3 inactivates the virulence factors of pathogens. The present results suggest that pathogenic differences in *Bordetella* are attributed to the different activities of adenylate cyclase toxins.

## INTRODUCTION

Bordetella pertussis causes whooping cough, a contagious respiratory disease that is recently resurgent despite high vaccination coverage (1, 2). Bordetella pertussis is often called “classical *Bordetella*,” together with closely related Bordetella parapertussis and Bordetella bronchiseptica, which also cause respiratory infections in various host animals with severe to mild coughing. A genomic analysis indicated that *B*. *pertussis* and *B*. *parapertussis* independently evolved from a B. bronchiseptica-like ancestor (3). Classical *Bordetella* is known to share many virulence factors, including adenylate cyclase toxin (CyaA), dermonecrotic toxin (DNT), and filamentous hemagglutinin, but not pertussis toxin, which is specific to *B*. *pertussis*. The molecular actions of these major virulence factors have been extensively examined. However, the pathogenesis of *Bordetella* infections has yet to be elucidated in detail (4). Furthermore, disease-causing propensities and host specificities apparently differ among classical *Bordetella* (5). *B*. *pertussis* is a strictly human pathogen that causes acute and severe respiratory infections with paroxysmal coughing and vomiting. *B*. *parapertussis* infects sheep and humans and is associated with a milder pertussis-like disease. *B*. *bronchiseptica* exhibits the broadest host range of mammals and leads to chronic and often asymptomatic infections. Limited information is currently available on how these three species exhibit different characteristics as pathogenic bacteria despite sharing many homologous virulence factors.

CyaA, one of the virulence factors commonly produced by classical *Bordetella*, is considered to play a key role in the establishment of infection (5). The toxin consists of two functional modules: the N-terminal adenylate cyclase domain (ACD), which is activated by eukaryotic calmodulin and causes supraphysiological cyclic AMP (cAMP) accumulation after being translocated into the cytosol of target cells, and the C-terminal RTX (repeats-in-toxin) domain, which is responsible for binding to target cells and organizing cation-selective toxin pores on the cell membrane (6). The toxin affects myeloid cells expressing CD11b/CD18 and subverts host immunity by inhibiting phagocytosis, chemotaxis, and superoxide production and modulating dendritic cell maturation and inflammatory cytokine/chemokine production (7–13). CyaA also lyses erythrocytes and induces apoptosis in macrophages (14–16). Moreover, previous studies showed that epithelial cells may be targets of CyaA (17, 18).

Since CyaA is highly conserved among classical *Bordetella*, its function and role have been considered to be similar in bacterial infections. However, we herein demonstrated that *B*. *bronchiseptica* CyaA, in contrast to *B*. *pertussis* CyaA, exhibited only weak toxicity in nucleated cells. After being translocated into target cells, *B*. *bronchiseptica* CyaA was phosphorylated in the cytosol and associated with the eukaryotic host factor 14-3-3, which resulted in the abrogation of adenylate cyclase activity. To the best of our knowledge, this is the first study to demonstrate that a virulence factor of pathogens is inactivated by 14-3-3 in target cells. Our results also suggest that a difference in cytotoxic effects between *B*. *pertussis* CyaA and *B*. *bronchiseptica* CyaA influences the pathogenicity of each *Bordetella* species in a rat infection model.

## RESULTS

### CyaA of B. pertussis, but not CyaA of B. bronchiseptica or B. parapertussis, induces morphological changes and cAMP accumulation in L2 cells.

**FIG 1:**
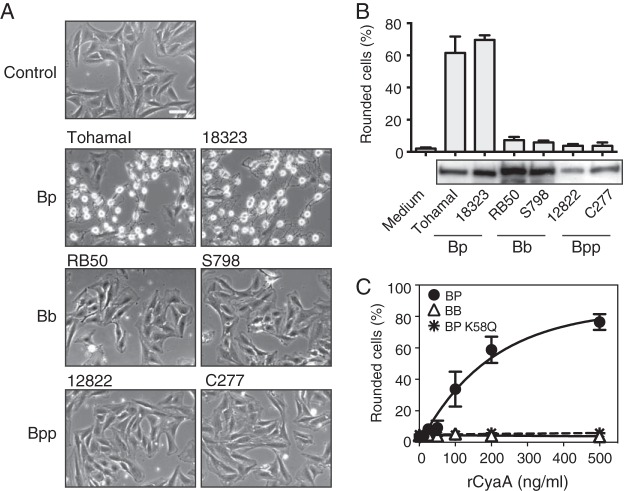
Cell-rounding activities of CyaAs in culture supernatants of Bordetella pertussis (Bp), B. bronchiseptica (Bb), and B. parapertussis (Bpp). (A) Morphological changes in L2 cells treated with the culture supernatant of each *Bordetella* species. Images were taken after 3-h incubation at 37°C after the addition of *Bordetella* culture supernatants to L2 cells. Bar, 50 µm. (B) The percentage of rounded L2 cells in panel A and immunoblotted using anti-CyaA antiserum to detect CyaA in the tested culture supernatant of each *Bordetella* species. The number of rounded cells was counted and represented as a percentage of the total number of cells in the microscopic fields (>100 cells/sample). Values are means plus standard deviations (SD) (error bars) (*n* = 4). (C) The percentage of rounding of L2 cells treated with each recombinant CyaA (rCyaA) at various concentrations. Values are means ± SD (*n* = 4).

**FIG 2:**
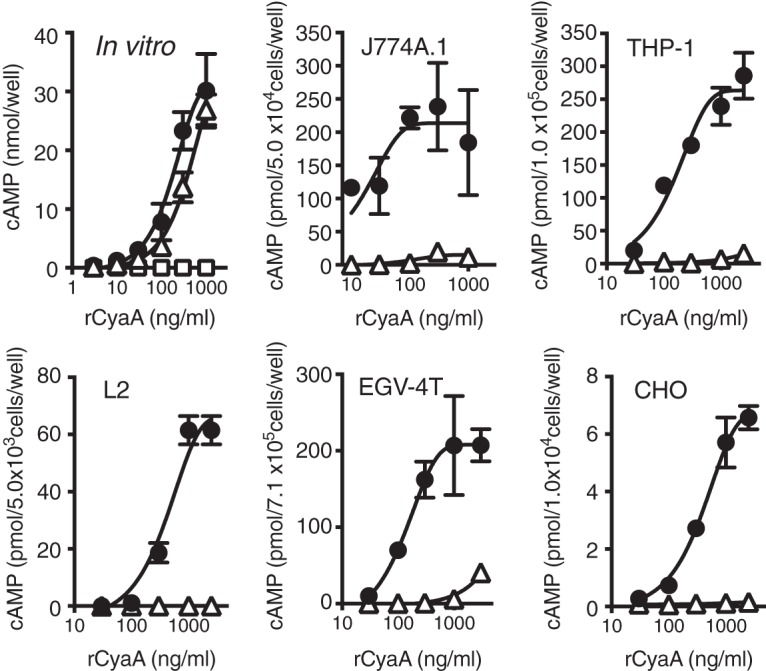
Adenylate cyclase activities of recombinant *B*. *pertussis* and *B*. *bronchiseptica* CyaA *in vitro* or in cultured cells. The adenylate cyclase activities of recombinant CyaAs (rCyaA) (*B*. *pertussis* CyaA [●], *B*. *bronchiseptica* CyaA [△], and *B*. *pertussis* CyaAK58Q [□]) were assessed *in vitro* or in cultured cells. The cultured cells, J774A.1 (mouse macrophage), THP-1 (human monocyte), L2 (rat type 2 alveolar epithelial cell), EGV-4T (rat tracheal epithelial cell), and CHO-K1 (Chinese hamster ovary cell) cells, were incubated at the indicated concentrations for appropriate periods in order to adhere to wells and then treated with CyaAs, as described in Text S1 in the supplemental material. Values are means ± standard deviations (SD) (error bars) (*n* = 3).

We previously reported that *B*. *pertussis* CyaA caused cell rounding in L2 cells through its adenylate cyclase activity (19): anti-CyaA serum neutralized cell-rounding activity of the culture supernatant of B. pertussis, indicating that CyaA (*B*. *pertussis* CyaA) is the sole cause of cell rounding. In contrast, we found that the culture supernatants of *B*. *bronchiseptica* and *B*.* parapertussis*, which contain CyaA, did not alter cell morphology (Fig. 1A and B; see Fig. S1A in the supplemental material). Ten strains each of *B*. *pertussis* and *B*. *bronchiseptica* that were maintained in the laboratory were similarly examined for their cell-rounding activities. Although the culture supernatants of *B*. *pertussis* strains caused cell rounding, a result that is consistent with previous findings (19), the culture supernatants of all the *B*. *bronchiseptica* strains tested did not (data not shown). These results indicate that a discrepancy in CyaA toxicity was commonly observed between *B*. *pertussis* and *B*. *bronchiseptica* but was not due to some unusual strains. When L2 cells were treated with purified preparations of recombinant CyaAs, similar results were obtained: *B*. *pertussis* CyaA induced cell rounding in a dose-dependent manner, while *B*. *bronchiseptica* CyaA did not at any of the concentrations tested (Fig. 1C). As previously reported (19), *B*. *pertussis* CyaA K58Q, which is defective in adenylate cyclase activity, did not cause cell rounding. We then examined the enzyme activities of *B*. *pertussis* CyaA and *B*. *bronchiseptica* CyaA to generate cAMP from ATP *in vitro*. Both CyaAs similarly produced cAMP, indicating that the enzyme activity of *B*. *bronchiseptica* CyaA is intact (Fig. 2). In contrast, when cultured cells were treated with the toxins, a marked increase in intracellular cAMP was observed only in *B*. *pertussis* CyaA-treated cells (Fig. 2). These results indicate that *B*. *bronchiseptica* CyaA is enzymatically active *in vitro*, but it does not appear to exhibit cytotoxicity that is mediated by the accumulation of intracellular cAMP.

10.1128/mBio.00628-18.2 FIG S1  CyaA preparations used in the present study. (A) Immunoblotting of CyaA in the culture supernatant of each *Bordetella* species. A complete image of the membrane corresponding to that in Fig. 1B is shown. (B) SDS-PAGE of the recombinant CyaAs used in the present study. The recombinant CyaAs were produced and purified as described in the supplemental Materials and Methods in Text S1. Proteins were applied at 1 µg/lane, and stained by Coomassie brilliant blue R-250. Recombinant CyaAs (rCyaA) were indicated by open arrowheads. Download FIG S1, EPS file, 2.3 MB. Copyright © 2018 Fukui-Miyazaki et al. 2018 Fukui-Miyazaki et al. This content is distributed under the terms of the Creative Commons Attribution 4.0 International license.

10.1128/mBio.00628-18.1 TEXT S1  Supplemental Materials and Methods and supplemental references. Download TEXT S1, DOCX file, 0.1 MB. Copyright © 2018 Fukui-Miyazaki et al. 2018 Fukui-Miyazaki et al. This content is distributed under the terms of the Creative Commons Attribution 4.0 International license.

### Phe^375^ is the key residue for the cytotoxicity of CyaA.

**FIG 3:**
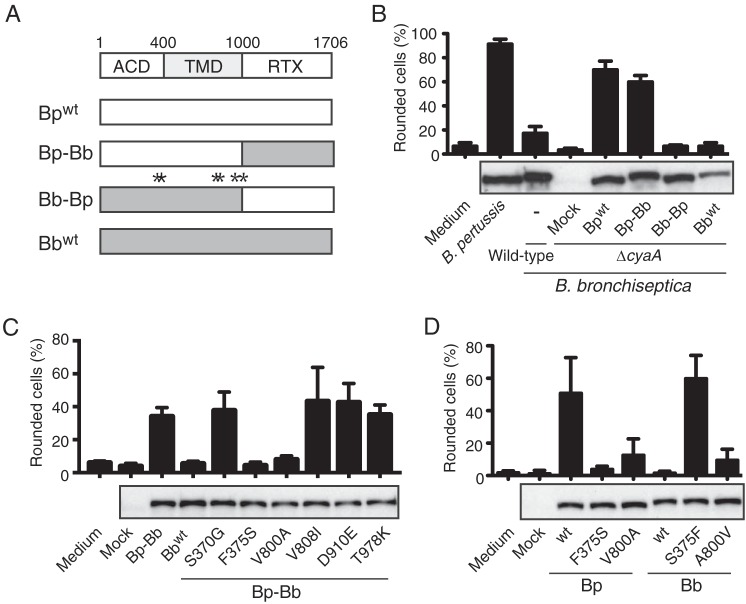
Identification of the amino acid residues crucial for the cytotoxic activity of *B*. *pertussis* CyaA. (A) Schematic representation of chimeric CyaAs. CyaA is composed of an adenylate cyclase domain (ACD), hydrophobic transmembrane domain (TMD), and RTX domain in order from the N terminus to the C terminus. Asterisks indicate the positions of six amino acid replacements in the ACD-TMD region between *B*. *pertussis* (Bp) and *B*. *bronchiseptica* (Bb) CyaA (see also Fig. S2 in the supplemental material). The numbers indicate the amino acid positions of the start and/or end of the domains. In the following experiments, B. pertussis Tohama I and B. bronchiseptica RB50 were used as the sources for CyaA genes, and constructed genes were introduced into B. bronchiseptica RB50 Δ*cyaA*, as described in Text S1. (B to D) Percentages of rounded L2 cells treated with the culture supernatants of wild-type (wt) *Bordetella* and derivatives: B. pertussis (Bp), B. bronchiseptica (Bb) or B. bronchiseptica Δ*cyaA* complemented with wild-type or each chimeric *cyaA* (B), B. bronchiseptica Δ*cyaA* complemented with each B. pertussis-*B*. *bronchiseptica* chimeric *cyaA* with or without a point mutation at the position of the amino acid replacement (C), B. bronchiseptica Δ*cyaA* complemented with *B*. *pertussis* or *B*. *bronchiseptica cyaA* with or without the mutation at position 375 or 800 (D). The sample from B. bronchiseptica Δ*cyaA* transformed with the empty vector was used as mock infection. Each CyaA in the culture supernatant was detected by immunoblotting with anti-CyaA antiserum Values are means plus SD (*n* = 4).

*B*. *bronchiseptica* CyaA and *B*. *pertussis* CyaA show 98% homology with 36 amino acid replacements in the overall sequence. In order to identify the crucial structural differences responsible for the different cytotoxic activities of *B*. *bronchiseptica* CyaA and *B*. *pertussis* CyaA, we initially attempted to localize the region of *B*. *pertussis* CyaA necessary for cell-rounding activity. We examined chimeric CyaAs, in which the RTX domains were mutually exchanged between *B*. *pertussis* CyaA and *B*. *bronchiseptica* CyaA, for their cell-rounding activities (Fig. 3A). B. pertussis-B. bronchiseptica chimeric CyaA with the B. bronchiseptica-derived RTX domain caused cell rounding as well as *B*. *pertussis* CyaA did, whereas *B*. *bronchiseptica* CyaA and B. bronchiseptica-*B*. *pertussis* chimeric CyaA with the B. pertussis-derived RTX domain did not (Fig. 3B), indicating that cell-rounding activity is dependent on the region, including the adenylate cyclase domain (ACD) and transmembrane domain (TMD) of *B*. *pertussis* CyaA.

10.1128/mBio.00628-18.3 FIG S2  Identification of the translation start site of *B*. *bronchiseptica* CyaA. (A) Amino acid sequences of the ACD-TMD region of *B*. *pertussis* (Bp) CyaA and B. bronchiseptica (Bb) CyaA. Met (+1) and Val (−34) had been annotated as the translation start sites of Bp and Bb CyaAs, respectively (10), which resulted in the N-terminal extension (colored blue) of Bb CyaA. The asterisks indicate the positions of the amino acid replacement between Bp CyaA and Bb CyaA. (B) Identification of the actual start codon for Bb CyaA. We induced mutations independently in *cyaA* of Bb at codons GTG for Val (−34) to GGG and ATG for Met (+1) to ATC, and introduced each mutated gene into Bb Δ*cyaA*. Each Bb was cultured for 6 h, and the culture supernatant and whole-cell lysates were subjected to immunoblotting for CyaA. The mutation at Met (+1), but not Val (−34), resulted in the abrogation of protein expression, indicating that the actual start codon for Bb CyaA is that for Met (+1), which is identical to that for Bp CyaA. Therefore, we concluded that the putative N-terminal extension does not exist in Bb CyaA. Bp^wt^ and Bb^wt^ plasmids carrying *cyaA* of Bp and Bb, respectively; Bb^−34mut^, *cyaA* with a mutation at the codon for Val (−34); Bb^1mut^, *cyaA* with a mutation at the codon for Met (+1). CyaA and FtsZ as a loading control were detected with anti-CyaA antiserum or an anti-FtsZ antibody prepared in our laboratory (7). Download FIG S2, EPS file, 0.6 MB. Copyright © 2018 Fukui-Miyazaki et al. 2018 Fukui-Miyazaki et al. This content is distributed under the terms of the Creative Commons Attribution 4.0 International license.

In the region including ACD and TMD (1 to 1,000 amino acids [aa]), there were 6 of the 36 amino acid replacements between *B*. *pertussis* CyaA and *B*. *bronchiseptica* CyaA (Fig. 3A and S2A). Although *B*. *bronchiseptica* CyaA has been reported to carry an N-terminal extension of 34 amino acid residues, whereas *B*. *pertussis* CyaA does not (3), we experimentally confirmed that this was not the case and that the translation start site of *B*. *bronchiseptica* CyaA was identical to that of *B*. *pertussis* CyaA (Fig. S2B). In order to identify which amino acid residue is crucial for the cytotoxicity of *B*. *pertussis* CyaA, we generated recombinant CyaAs by replacing the amino acid residue of the chimeric B. pertussis-B. bronchiseptica CyaA with that of *B*. *bronchiseptica* CyaA at each position and then examined their cell-rounding activities (Fig. 3C and S2A). The recombinant CyaAs F375S and V800A, the amino acids of which were exchanged with those of *B*. *bronchiseptica* CyaA at amino acid positions 375 and 800, respectively, failed to cause cell rounding, whereas the activities of other recombinant CyaAs were intact. We generated another series of recombinant CyaAs by exchanging the amino acid at position 375 or 800 between *B*. *bronchiseptica* CyaA and *B*. *pertussis* CyaA and again examined cell-rounding activity. The *B*. *pertussis* CyaA mutants F375S and V800A both lost their cell-rounding activities (Fig. 3D), indicating that Phe^375^ and Val^800^ are essential for the cell-rounding activity of *B*. *pertussis* CyaA. In contrast, the replacement of Ser with Phe at position 375 made *B*. *bronchiseptica* CyaA active on cells, while the mutant CyaA A800V remained inactive. These results indicate that the amino acid at position 375 is responsible for the different cytotoxic activities of CyaAs between *B*. *pertussis* and B. bronchiseptica: CyaA with Phe^375^ (*B*. *pertussis* CyaA) is active on cells, whereas the activity of CyaA with Ser^375^ (*B*. *bronchiseptica* CyaA) was almost negligible. The results of *in vitro* infection assays supported these results: wild-type B. pertussis and the B. bronchiseptica mutant strain producing *B*. *bronchiseptica* CyaA_S375F_ markedly increased intracellular cAMP levels in L2 and J774A.1 cells, whereas wild-type B. bronchiseptica and the B. pertussis mutant strain producing *B*. *pertussis* CyaA_F375S_ barely increased intracellular cAMP (Fig. S3). We checked the amino acid residues at position 375 of CyaAs, the sequences of which are deposited in the genome database of the National Center for Biotechnology Information (NCBI), and found that all *B*. *pertussis* (543 deposited data sets) and *B*. *bronchiseptica* (87 deposited data sets) carried Phe^375^ and Ser^375^, respectively. In contrast, Val^800^ was conserved in *B*. *pertussis*, whereas Ala^800^ was not conserved in *B*. *bronchiseptica*: 43 out of 87 deposited data sets of *B*. *bronchiseptica* CyaA showed Ala^800^, whereas the others showed Val^800^. These results indicate that the 375th amino acid replacement is phylogenetically conserved, and Val^800^ appears to be involved in the cytotoxic activity of *B*. *pertussis* CyaA for unknown reasons. Thus, we focused on the amino acid residue at position 375.

10.1128/mBio.00628-18.4 FIG S3  Intracellular cAMP in cultured cells infected with Bordetella pertussis, B. bronchiseptica, and their mutants producing CyaA derivatives. L2 cells (A) or J774A.1 cells (B) were infected at a multiplicity of infection (MOI) of 100 or 20, respectively, with B. pertussis (Bp) or B. bronchiseptica (Bb) wild-type (WT) or mutant strains producing CyaA with the 375th amino acid replacement (Bp F375S and Bb S375F), and *cyaA*-deficient (Δ*cyaA*) strains, as described in the supplemental Materials and Methods in Text S1. The results for B. pertussis-infected or B. bronchiseptica-infected cells are indicated as filled or open columns, respectively. Each bar represents the mean ± SD (*n* = 3). Data were statistically analyzed by a one-way ANOVA with Tukey’s multiple-comparison test. *, *P* < 0.001; **, no significant differences. Download FIG S3, EPS file, 0.4 MB. Copyright © 2018 Fukui-Miyazaki et al. 2018 Fukui-Miyazaki et al. This content is distributed under the terms of the Creative Commons Attribution 4.0 International license.

### *B. bronchiseptica* CyaA is not defective in each intoxication step on target cells.

**FIG 4:**
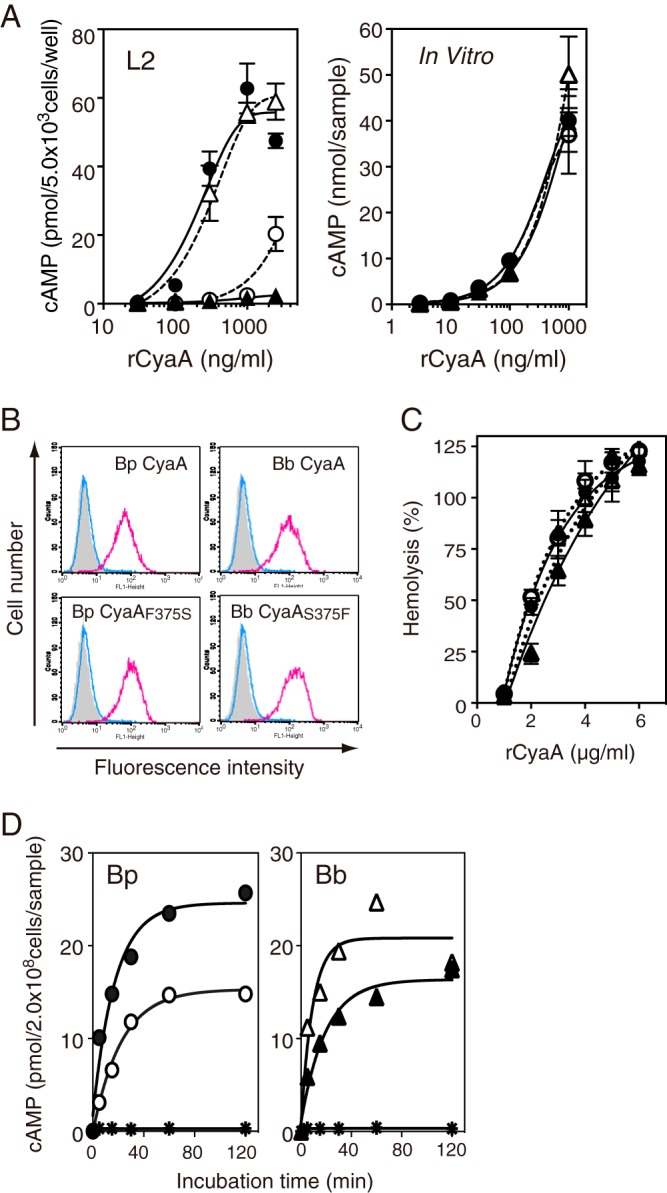
Toxic actions of CyaA and its derivatives in each intoxication step. (A) The adenylate cyclase activities of *B*. *pertussis* (Bp) CyaA (●), *B*. *pertussis* CyaA_F375S_ (○), *B*. *bronchiseptica* (Bb) CyaA (▴), and *B*. *bronchiseptica* CyaA_S375F_ (△) were assessed in L2 cells and *in vitro*. Values are means ± SD (*n* = 3). (B) Binding of CyaA to L2 cells, as detected by flow cytometry with an anti-CyaA polyclonal antibody (red line) or normal (healthy) rabbit antibody (blue line). (C) Hemolytic activity of CyaA on sheep erythrocytes. (D) Translocation of the enzyme domain of CyaA. cAMP production in sheep erythrocytes, which have no specific receptor for CyaA, was assessed after treatment with each CyaA preparation. Asterisks indicate the values for samples without CyaA. (C and D) Each symbol represents the value for the test group corresponding to the symbols shown in panel A.

**FIG 5:**
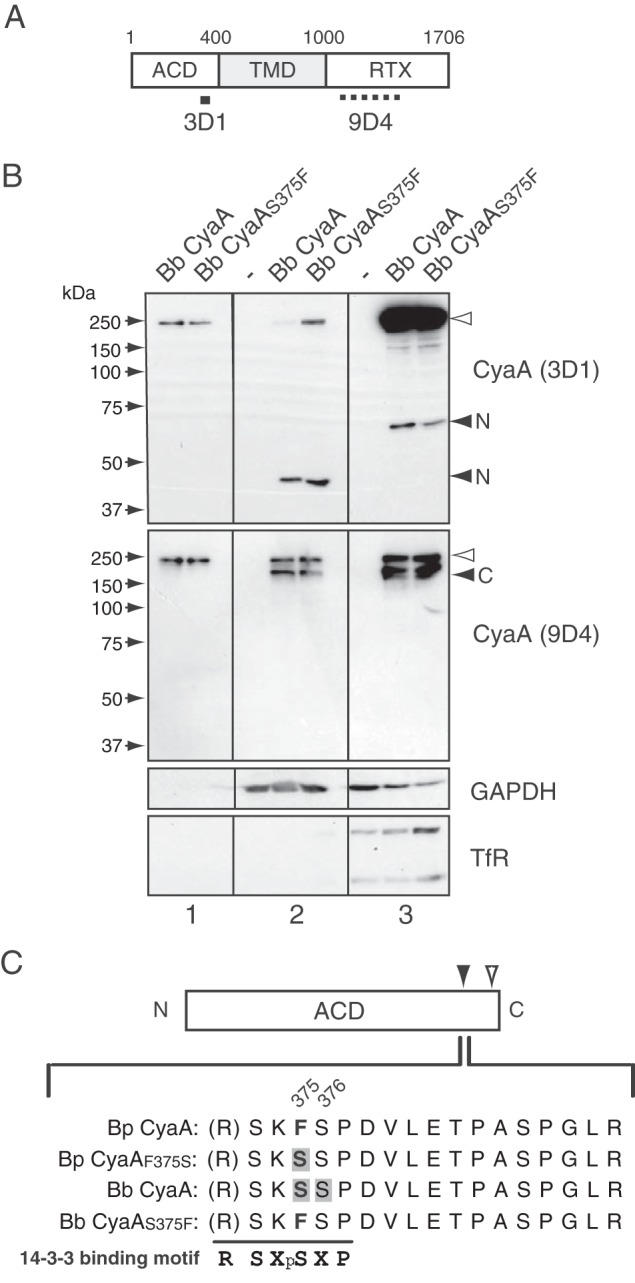
Mass spectrometric analysis of the adenylate cyclase domain (ACD) liberated in the cytosol. (A) Schematic representation of CyaA. The regions recognized by the anti-CyaA monoclonal antibodies 3D1 and 9D4 are indicated by solid and dotted lines, respectively (56). (B) Immunoblotting of the cytosolic and membrane fractions of J774A.1 cells treated with *B*. *bronchiseptica* (Bb) CyaA and *B*. *bronchiseptica* CyaA_S375F_. Cell fractions were prepared as described in Materials and Methods and separated by SDS-PAGE after trichloroacetic acid (TCA) precipitation, followed by immunoblotting with an anti-CyaA monoclonal antibody (3D1 or 9D4), anti-GAPDH antibody, and anti-transferrin receptor (anti-TfR) antibody. The positions of N- and C-terminal fragments and the full-length CyaA are indicated by black and white arrowheads, respectively. Panels 1, CyaA preparations (2 ng/lane); panels 2, cytosol fractions (100 µg/lane); panels 3, membrane fractions (10 µg/lane). (C) Localization of phosphorylated amino acid residues in ACD isolated from the cytosolic fraction of J774A.1 cells treated with each CyaA. All CyaAs were phosphorylated at S^393^ (white arrowhead). The black arrowhead indicates the additional phosphorylation site that is specific to *B*. *bronchiseptica* (Bb) CyaA and *B*. *pertussis* (Bp) CyaA_F375S_. The sequences of isolated peptides surrounding the phosphorylation site are shown at the bottom of the panel. Phosphorylated serines (S^375^ or S^376^) are shown on a gray background in the sequences with the numbers of the amino acid positions. The 14-3-3 binding motif was aligned below the sequences. X, any type of residue; pS, phosphorylated serine.

In order to elucidate the reason why *B*. *bronchiseptica* CyaA does not exert cytotoxic activity, we examined purified preparations of *B*. *bronchiseptica* CyaA, *B*. *pertussis* CyaA, *B*. *bronchiseptica* CyaA_S375F_, and *B*. *pertussis* CyaA_F375S_ for actions in each intoxication step (Fig. 4 and Fig. S1B). Following their addition to a culture of L2 cells, *B*. *pertussis* CyaA and *B*. *bronchiseptica* CyaA_S375F_ increased intracellular cAMP levels, whereas *B*. *bronchiseptica* CyaA and *B*. *pertussis* CyaA_F375S_ did not, which is consistent with the results of the cell-rounding assay (Fig. 3D and 4A). However, the *in vitro* enzyme activities of these CyaA preparations were intact (Fig. 2 and 4A). The actions of CyaA on target cells are at least divided into the following steps (6): (i) binding to cells; (ii) translocation of the enzyme domain into the cytosol; (iii) formation of toxin pores to make the plasma membrane permeable; and (iv) exertion of enzymatic activity. Therefore, we examined CyaA preparations for each intoxication step. A flow cytometric analysis showed that all CyaA preparations similarly bound to L2 cells (Fig. 4B). The formation of toxin pores and translocation of the enzyme domain have been reported to occur in an unrelated manner after toxin binding (20). All CyaA preparations showed equivalent hemolytic activities on sheep erythrocytes (Fig. 4C). Translocation of the enzyme domain, as previously estimated by increases in cAMP levels in sheep erythrocytes (21), was confirmed for all CyaA preparations, although *B*. *pertussis* CyaA_F375S_ and *B*. *bronchiseptica* CyaA were less efficient than *B*. *pertussis* CyaA and *B*. *bronchiseptica* CyaA_S375F_ (Fig. 4D). *B*. *pertussis* CyaA has been reported to undergo proteolytic cleavage in the cytosol and liberate fragments of ~45 kDa, including the enzyme domain (22). After the treatment of J774A.1 cells with *B*. *bronchiseptica* CyaA and *B*. *bronchiseptica* CyaA_S375F_, we detected 46-kDa fragments in the cytosol using the 3D1 antibody, which recognizes the enzyme domain (Fig. 5A and B). Similar results were obtained in experiments with *B*. *pertussis* CyaA and *B*. *pertussis* CyaA_F375S_ (data not shown). These results demonstrate that *B*. *bronchiseptica* CyaA and *B*. *pertussis* CyaA_F375S_ carrying Ser^375^, as well as *B*. *pertussis* CyaA and *B*. *bronchiseptica* CyaA_S375F_ carrying Phe^375^, are not defective in any intoxication steps against target cells.

### CyaA with Ser^375^ interacts with 14-3-3 after phosphorylation.

In the above-described results of immunoblotting (Fig. 5B for *B*. *bronchiseptica* CyaA and *B*. *bronchiseptica* CyaA_S375F_), we noted that the fragment containing ACD of *B*. *bronchiseptica* CyaA and *B*. *pertussis* CyaA_F375S_ showed slightly slower mobility on SDS-polyacrylamide gels than *B*. *pertussis* CyaA and *B*. *bronchiseptica* CyaA_S375F_, implying that S^375^ in the fragment underwent some covalent modification in the cytosol of target cells. Mass spectrometric analyses revealed that 46-kDa fragments of *B*. *bronchiseptica* CyaA and *B*. *pertussis* CyaA_F375S_, which were composed of at least ~399 (~R^399^) amino acid residues covering the enzyme domain, were phosphorylated at Ser^375^ (Fig. 5C).

**FIG 6:**
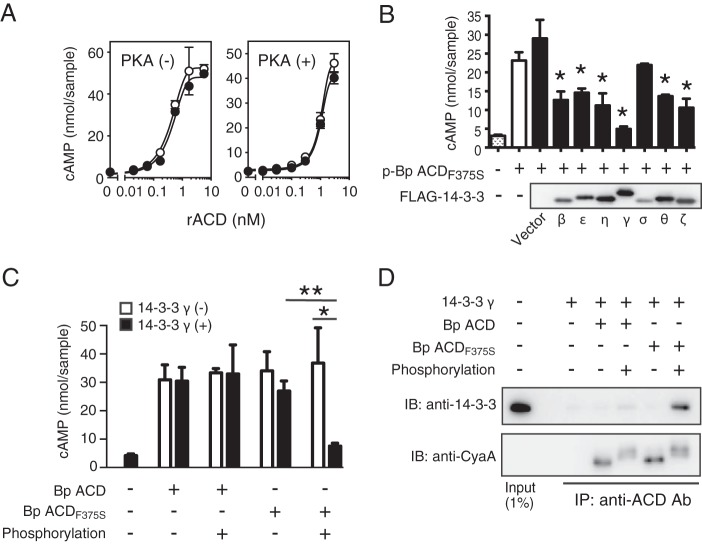
Inactivation of phosphorylated *B*. *pertussis* ACD_F375S_ by 14-3-3. (A) *In vitro* enzyme activities of *B*. *pertussis* ACD (●) and *B*. *pertussis* ACD_F375S_ (○) with (+) or without (-) phosphorylation by the protein kinase A (PKA) treatment. Values are means ± SD (error bars) (*n* = 3). rACD, recombinant ACD. (B) Inhibition of the enzyme activity of phosphorylated *B*. *pertussis* ACD_F375S_ (p-Bp ACD_F375S_) in the presence of the 14-3-3 proteins expressed in E. coli. p-Bp ACD_F375S_ (1 nM) was preincubated with 10 µl of the FLAG-14-3-3-containing E. coli fraction (~50 µg of total protein) at 37°C for 30 min before measuring enzyme activity *in vitro*. The expression of each isoform was confirmed by immunoblotting with an anti-FLAG antibody. Value are means plus SD (*n* = 3). The statistical significance of differences was analyzed by an unpaired *t* test. Values that are significantly different (*P* < 0.01) from the value for the control “vector” are indicated by an asterisk. (C) Phosphorylation-dependent inactivation of the enzyme activities of *B*. *pertussis* ACDs by 14-3-3γ. *B*. *pertussis* (Bp) ACD (1 nM)and *B*. *pertussis* ACD_F375S_ (1 nM) were phosphorylated by PKA as described in Materials and Methods and preincubated with 48 µg of the FLAG-14-3-3γ-containing E. coli fraction before measuring enzyme activity *in vitro*. Values are means pus SD (*n* = 3). The statistical significance of differences was analyzed by an unpaired *t* test and indicated by asterisks as follows: *, *P* < 0.05; **, *P* < 0.001. (D) Immunoprecipitation assay for the interaction between ACDs and 14-3-3γ. The reaction solutions in panel C were immunoprecipitated (IP) using an anti-ACD antibody (Ab), followed by immunoblotting (IB) with an anti-14-3-3 antibody and anti-CyaA antibody (3D1).

In order to examine whether the phosphorylation of Ser^375^ influences the toxic action of CyaA on target cells, we prepared recombinant ACD peptides of *B*. *pertussis* CyaA (*B*. *pertussis* ACD) and *B*. *pertussis* CyaA_F375S_ (*B*. *pertussis* ACD_F375S_) phosphorylated by pretreatment with protein kinase A (PKA) *in vitro* and examined them for adenylate cyclase activity. *B*. *pertussis* ACD and *B*. *pertussis* ACD_F375S_, which consist of the region from amino acids at positions 2 to 400, were both phosphorylated with PKA, as judged by mobility shifts in SDS-polyacrylamide gels with Phos-tag and mass spectrometry (Fig. S4). The *in vitro* adenylate cyclase activities of these ACDs were unchanged before and after phosphorylation, indicating that the phosphorylation of Ser^375^ *per se* does not influence the enzyme activity of CyaA (Fig. 6A).

10.1128/mBio.00628-18.5 FIG S4  Phosphorylation of ACDs. (A) SDS-PAGE of ACDs of *B*. *pertussis* CyaA (Bp ACD) and B. pertussis CyaA_F375S_ (Bp ACD_F375S_). The purified preparations of each sample were applied to SDS-polyacrylamide gels at 1 µg/lane and stained by Coomassie brilliant blue R-250 after electrophoresis. (B) Phosphorylation of Bp ACDs. Bp ACD and Bp ACD_F375S_ were phosphorylated by PKA, as described in Materials and Methods, and subjected to phosphate affinity SDS-PAGE with 50 µM Phos-tag (Wako, Osaka, Japan), followed by immunoblotting with an anti-ACD antibody according to the manufacturer’s instructions. In phosphate affinity SDS-PAGE, phosphorylated proteins move more slowly than nonphosphorylated proteins. (C) Localization of phosphorylated amino acid residues in ACDs treated with PKA. White and black arrowheads indicate the positions of phosphorylated amino acids in phosphorylated Bp ACD (p-Bp ACD) and Bp ACD_F375S_ (p-Bp ACD_F375S_): p-Bp ACD was phosphorylated at S^218^, S^227^, and S^393^, and p-Bp ACD_F375S_ was additionally phosphorylated at S^373^, S^375^, or S^376^. The amino acid sequences of the regions surrounding the 375th residue are also shown, with the shaded boxes indicating the positions of phosphorylation. Download FIG S4, EPS file, 0.7 MB. Copyright © 2018 Fukui-Miyazaki et al. 2018 Fukui-Miyazaki et al. This content is distributed under the terms of the Creative Commons Attribution 4.0 International license.

We then searched a phosphorylation-related motif in ACD carrying Ser^375^ with Scansite 3 (http://scansite3.mit.edu/) and found that the region surrounding phosphorylated Ser^375^ corresponded to a 14-3-3 binding motif (mode 1, RSXpS/pTXP, in which pS and pT represent phosphoserine and phosphothreonine, respectively, and X is any type of residue [Fig. 5C]) (23, 24). The 14-3-3 protein family comprises seven isoforms that are expressed ubiquitously in a wide range of eukaryotic organisms and are known to regulate phosphoserine/phosphothreonine-mediated intracellular signal transduction by interacting with a number of phosphorylated proteins (23, 25). Therefore, we examined the influence of 14-3-3 on the enzyme activity of *B*. *pertussis* ACD_F375S_ phosphorylated with PKA and found that each isoform of the 14-3-3 protein, except for the σ isoform, significantly inhibited the *in vitro* adenylate cyclase activity of phosphorylated *B*. *pertussis* ACD_F375S_ to various extents (Fig. 6B). In contrast to phosphorylated *B*. *pertussis* ACD_F375S_, the activities of *B*. *pertussis* ACD and nonphosphorylated *B*. *pertussis* ACD_F375S_ were not influenced by 14-3-3 (Fig. 6C). These results indicate that the inhibition of adenylate cyclase activity was dependent on the phosphorylation of Ser^375^. The immunoprecipitation assay revealed that 14-3-3γ interacted with phosphorylated, but not nonphosphorylated, *B*. *pertussis* ACD_F375S_. The apparent interaction between *B*. *pertussis* ACD and 14-3-3γ was not detected regardless of the phosphorylation state (Fig. 6D). Similar results were obtained when the cytosolic fraction of J774A.1 cells, which contained 14-3-3, was used instead of *Escherichia coli*-produced recombinant 14-3-3γ (Fig. S5). These results indicate that the activity of CyaA with Ser^375^ was inhibited by 14-3-3 of the target cells after the phosphorylation of Ser^375^.

10.1128/mBio.00628-18.6 FIG S5  Inactivation of phosphorylated B. pertussis (Bp) ACD_F375S_ by the cytosolic fraction of J774A.1 cells. (A) Reverse transcription-PCR (RT-PCR) analysis of 14-3-3 isoforms in J774A.1 cells. The results obtained indicate that J774A.1 cells express all isoforms of the 14-3-3 family. The primers used are listed in Table S2. (B) Phosphorylation-dependent inactivation of ACDs in the presence of the cytosolic fraction of J774A.1 cells. Each bar represents the mean ± SD (*n* = 3). The significance of differences was analyzed by an unpaired *t* test. *, *P* < 0.05; **, *P* < 0.005. (C) Immunoprecipitation assay for the interaction between Bp ACDs and 14-3-3 in the cytosol of J774A.1 cells. The reaction solutions in panel B were immunoprecipitated (IP) using an anti-ACD antibody, followed by immunoblotting (IB) with an anti-14-3-3 antibody or anti-CyaA antibody (3D1). Since the nonspecific binding of 14-3-3 was observed, even in the sample without ACD, the relative intensity of each band to the nonspecific band is shown below the top panel. The representative results of three independent experiments are shown. Download FIG S5, EPS file, 1.3 MB. Copyright © 2018 Fukui-Miyazaki et al. 2018 Fukui-Miyazaki et al. This content is distributed under the terms of the Creative Commons Attribution 4.0 International license.

### *B. pertussis* CyaA, but not *B. bronchiseptica* CyaA, disrupts epithelial barrier function.

**FIG 7:**
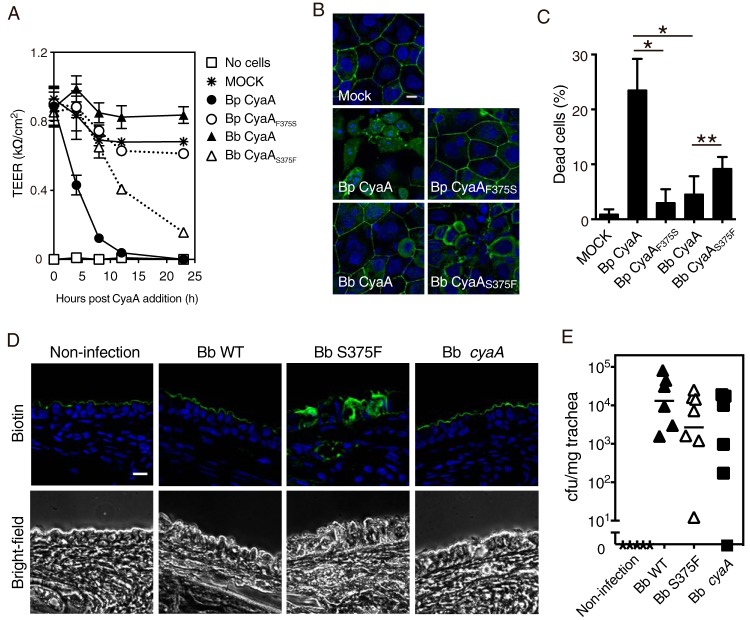
Disruption of epithelial barrier function by CyaA with Phe^375^, but not with Ser^375^. (A) Alterations in TEER of EGV-4T cells treated with CyaAs. Values are means ± SD (error bars) (*n* = 6). Bp, B. pertussis; Bb, B. bronchiseptica*.* (B) Staining of ZO-1 (green) and the nucleus (blue) in EGV-4T cells treated with CyaAs for 23 h. Untreated cells are labeled mock in the figure. Bar, 10 µm. (C) Percentage of dead EGV-4T cells grown on the Transwell insert after the 24-h treatment with CyaAs. Cells were counted in eight fields, and the percentage of dead or dying cells to the total number of cells (>100 cells) is shown. Values are means plus SD (*n* = 8). Data were statistically analyzed by a one-way analysis of variance (ANOVA) with Tukey’s multiple-comparison test. Values that are significantly different are indicated by a bar and asterisks as follows: *, *P* < 0.001; **, *P* = 0.0615. (D) Fluorescence staining for the tracheal-epithelial barrier structure in rats infected with wild-type B. bronchiseptica (Bb WT), *B*. *bronchiseptica* producing *B*. *bronchiseptica* CyaA_S375F_ (Bb S375F), and *cyaA*-deficient *B*. *bronchiseptica* (Bb Δ*cyaA*). Longitudinal sections of the trachea excised from *Bordetella*-infected rats on day 3 postinoculation were stained for the robustness of the epithelial barrier (green) and cell nucleus (blue). Details of the methods used are described in Materials and Methods. Experiments were repeated three times, and representative results are shown. Bar, 10 µm. (E) The numbers of bacteria recovered from the tracheas were measured on day 5 postinoculation. Each symbol represents the value for an individual rat. Each horizontal bar represents the geometric mean for a group of rats. The geometric mean is missing for the *B*. *bronchiseptica* Δ*cyaA* group because bacteria were not recovered from one specimen of the experimental group.

CyaA targets CD11b-expressing myeloid cells, such as macrophages, neutrophils, and dendritic cells, and subverts the immune responses of hosts by inhibiting bactericidal activities and affecting cytokine secretion (8, 9, 11–13). The cAMP-elevating activity of CyaA was previously reported to up- and downregulate the induction of interleukin 6 (IL-6) and tumor necrosis factor alpha (TNF-α), respectively (13, 17, 26). Therefore, we compared the effects of *B*. *pertussis* CyaA and *B*. *bronchiseptica* CyaA on the expression of IL-6 and TNF-α in L2 and NR8383 (rat alveolar macrophage) cells using real-time quantitative reverse transcription-PCR but observed no significant differences between *B*. *pertussis* CyaA and *B*. *bronchiseptica* CyaA, which is consistent with previous findings (13, 17, 26). This may have been because a slight increase in intracellular cAMP by residual active *B*. *bronchiseptica* CyaA was sufficient to affect cytokine production (note that *B*. *bronchiseptica* CyaA shows residual activity at higher concentrations, as shown in Fig. 2). We examined the effects of the toxin on epithelial cells, which were reportedly sensitive to CyaA (17, 18). Since previous studies indicated that CyaA does not exert clear cytotoxic (cell killing) activity against epithelial cells (17), we investigated whether CyaA affects the organization of the epithelial barrier, which is one of the important roles of the epithelium. Rat tracheal-epithelial EGV-4T cells were allowed to organize the epithelial barrier, as confirmed by high transepithelial electrical resistance (TEER) values, and were then treated with CyaA preparations applied to the apical side. The TEER values of *B*. *pertussis* CyaA and *B*. *bronchiseptica* CyaA_S375F_ decreased over time, whereas the values of *B*. *bronchiseptica* CyaA and *B*. *pertussis* CyaA_F375S_ did not (Fig. 7A). Immunofluorescence microscopy revealed that the structure of tight junctions visualized by anti-ZO-1 antibody was destroyed by treatment with *B*. *pertussis* CyaA and *B*. *bronchiseptica* CyaA_S375F_, but not by treatment with *B*. *bronchiseptica* CyaA and *B*. *pertussis* CyaA_F375S_ (Fig. 7B). In addition, *B*. *pertussis* CyaA, but not *B*. *bronchiseptica* CyaA, partly caused cell death under these experimental conditions (Fig. 7C). Consistent results were also obtained in rat infection experiments, in which rats were inoculated intranasally with a low dose (500 to 700 CFU) of B. bronchiseptica producing *B*. *bronchiseptica* CyaA (wild-type B. bronchiseptica [Bb WT]) or *B*. *bronchiseptica* CyaA_S375F_ (Bb S375F) (Fig. 7D). On day 3 postinoculation, the tracheas, the epithelia of which were labeled with *N*-hydroxysuccinimide (NHS)-linked biotin that was introduced into the tracheal lumen, were excised and subjected to microscopy (Fig. 7D). The infection with wild-type (WT) *B*. *bronchiseptica* and *B*. *bronchiseptica* Δ*cyaA* did not disrupt the barrier function of the tracheal epithelium, as judged by the intact apical area labeled with biotin. In contrast, in rats infected with *B*. *bronchiseptica* S375F, the introduced biotin permeated the submucosal layers through the epithelium, indicating that *B*. *bronchiseptica* CyaA_S375F_ impaired the barrier function of the tracheal epithelium (Fig. 7D). As shown in Fig. 7E, all bacteria tested colonized the rat trachea to similar extents.

## DISCUSSION

The CyaAs of classical *Bordetella* are highly homologous: *B*. *pertussis* CyaA shows 97.8 and 97.7% amino acid identities to *B*. *bronchiseptica* CyaA and *B*. *parapertussis* CyaA, respectively, while *B*. *bronchiseptica* CyaA shows 99.8% amino acid identity to *B*. *parapertussis* CyaA. Although most amino acid replacements between the CyaAs of classical *Bordetella* were found in the C-terminal region, which binds to the cell receptor and elicits protective immunity (27, 28), differences in the toxic activities of CyaAs were not reported (29, 30). In the present study, we demonstrated that the strength of the intracellular enzyme activity of *B*. *bronchiseptica* CyaA was markedly less than that of *B*. *pertussis* CyaA. *B*. *bronchiseptica* CyaA was enzymatically active *in vitro* but was inactivated by phosphorylation and a subsequent association with 14-3-3 in target cells. These results are not in conflict with the previous study showing an intracellular increase in cAMP levels after the treatment of cells with *B*. *bronchiseptica* CyaA (31), because our results also demonstrated that the intracellular enzyme activity of *B*. *bronchiseptica* CyaA was not completely abrogated (e.g., in J774A.1, THP-1, and EGV-4T cells in Fig. 2 and in J774A.1 cells in Fig. S3 in the supplemental material]). In addition, we found that *B*. *bronchiseptica* CyaA increased intracellular cAMP in erythrocytes (Fig. 4D), and this may have been because of the weak expression of 14-3-3 proteins (32) or unfavorable conditions for phosphorylation in erythrocytes. The amount of CyaA that translocated into the cytosol may also influence the efficiency of inactivation by 14-3-3. Furthermore, differences in responses to *B*. *bronchiseptica* CyaA among various cell types may occur due to different cell conditions as described above.

Ser^375^ of *B*. *bronchiseptica* CyaA was the crucial phosphorylation site responsible for the 14-3-3 association. NetPhos 3.1 (http://www.cbs.dtu.dk/services/NetPhos-3.1/) (33, 34) predicted PKA and CDC2 as possible kinases to phosphorylate Ser^375^. However, the types of kinases actually involved in the intracellular phosphorylation of Ser^375^ have not yet been identified, although PKA phosphorylated Ser^375^ of ACD *in vitro*. *B*. *pertussis* CyaA carries Phe^375^ instead of Ser^375^ and therefore is not phosphorylated or inactivated by 14-3-3. *B*. *parapertussis* CyaA, which has Ser^375^, did not cause cell rounding in L2 cells, indicating that it is also inactivated in an identical manner to *B*. *bronchiseptica* CyaA. A previous study showed that *B*. *pertussis* CyaA was cytotoxic against macrophages, whereas *B*. *parapertussis* CyaA was not (35), which is consistent with the present results.

14-3-3 proteins bind to the phosphoserine/threonine-containing sequence motifs of various target proteins and modulate their functions in diverse manners as follows: alterations in the intracellular localization or ability of target proteins to interact with other partners, the direct augmentation or inhibition of target protein activity, the protection of target proteins from proteolysis or dephosphorylation, and serving as a scaffold to bridge two distinct target molecules (24, 36, 37). The region of CyaA corresponding to the 14-3-3 motif (Arg^372^–Pro^377^) is not directly involved in enzyme activity or the association with calmodulin (38). However, the binding of 14-3-3 may interrupt the interaction between ACD and calmodulin because this region presumably faces the calmodulin binding space according to the crystal structures of the ACD-calmodulin complex (38). Alternatively, 14-3-3 may interfere with the conversion of ACD from the inactive state to the active state in response to calmodulin binding because the switch region of CyaA (Val^343^–Ala^364^) responsible for catalytic activation is sterically in the vicinity of the Arg^372^–Pro^377^ region (38). 14-3-3, which is known to regulate various biological processes, including mitogenic signal transduction, apoptotic cell death, and cell cycle control, has been reported to interact with hundreds of proteins, including Raf-1, tyrosine and tryptophan hydroxylases, Bcr, and Bad (25, 36, 37, 39–41). Regarding the virulence factors of pathogens, a previous study reported that Pseudomonas aeruginosa ExoS required 14-3-3 to exert its ADP-ribosylating effects (42). However, B. pertussis/B. parapertussis CyaAs are the first examples of the virulence factors of pathogens that are inactivated by 14-3-3.

Ser^375^ and Phe^375^ were found to be phylogenetically conserved in *B*. *bronchiseptica*/B. parapertussis and B. pertussis, respectively, implying that the different toxicities of CyaAs may be related to the distinct pathogenesis of *Bordetella*. In the present study, we compared the adenylate cyclase-derived toxicities of *B*. *bronchiseptica* CyaA and *B*. *pertussis* CyaA in relation to bacterial pathogenesis and found that CyaA with Phe^375^ (*B*. *pertussis* CyaA and *B*. *bronchiseptica* CyaA_S375F_), but not with Ser^375^ (*B*. *bronchiseptica* CyaA and *B*. *pertussis* CyaA_F375S_) disrupted tracheal epithelial barrier function. Cholera toxin (CT) and Bacillus anthracis edema factor (EF) were also recently revealed to disrupt host cell barrier function by inhibiting Rab11-mediated endocytic recycling, which is essential for transporting junctional proteins at tight/adherens junctions (43, 44). These effects were mediated by increases in intracellular cAMP induced by CT and EF. The mechanism by which CyaA with Phe^375^ disrupted the epithelial cell barrier may be similar to that by CT and EF. We also found that some cells in the confluent cell layer were killed by the treatment with CyaA with Phe^375^ (Fig. 7C), indicating that a long stimulation of active CyaA may induce epithelial cell death, which may exacerbate the disruption of the epithelial barrier. Similar epithelial damage was observed in rat tracheas infected with B. bronchiseptica expressing *B*. *bronchiseptica* CyaA_S375F_, but not *B*. *bronchiseptica* CyaA (Fig. 7D). These results imply that *B*. *pertussis* CyaA causes more extensive damage to the respiratory epithelium than *B*. *bronchiseptica*/B. parapertussis CyaA. This may explain the less severe clinical manifestations in *B*. *bronchiseptica* and *B*. *parapertussis* infections than in *B*. *pertussis* infections. However, it is important to note that CyaA plays a diverse role in *Bordetella* infections through several properties, such as adenylate cyclase activity, pore-forming activity, and potent immunogenicity (45–50), and only adenylate cyclase activity was disturbed by 14-3-3 in CyaAs of *B*. *bronchiseptica* and *B*. *parapertussis*. Furthermore, *B*. *bronchiseptica* CyaA modulated the production of IL-6 and TNF-α, similar to *B*. *pertussis* CyaA (data not shown), indicating that slight increases in cAMP are sufficient to induce biological effects in some cases. Thus, our results do not deny the roles of *B*. *bronchiseptica* CyaA as a virulence factor in B. bronchiseptica infection.

Although classical *Bordetella* share major virulence factors, they vary in disease severity and host specificity. The exact nature of this difference in pathogenesis remains unknown; however, previous studies pointed out distinct virulence factors produced by each *Bordetella* species, including pertussis toxin specific for B. pertussis, the type III secretion system apparently expressed in *B*.* bronchiseptica*, and the different lipopolysaccharide structures of each *Bordetella* species (45, 51–53). In addition to specific virulence factors, homologous ones may also play different roles in each *Bordetella* infection. We previously demonstrated that the expression level of DNT varied among classical *Bordetella* because of polymorphisms in the promoter region for the *dnt* gene (54). Most pig isolates of *B*. *bronchiseptica* carried the promoter types with increasing transcription activities, whereas human isolates of *B*. *pertussis* and *B*. *parapertussis* carried the least active promoter. These results appear to reflect the important role of DNT in the pathogenesis of pig infections with *B*. *bronchiseptica*. In the present study, we showed that CyaA may function differently as a virulence factor between *B*. *bronchiseptica*/B. parapertussis and *B*. *pertussis* infections. Studies on functional differences in homologous virulence factors, in addition to specific factors, may provide insights into the underlying reasons for the different pathogenicities and host specificities of classical *Bordetella*.

### Note.

After the manuscript had been completed, Hasan et al. reported the disruption of paracellular barrier function in human bronchial epithelial VA10 cells by *B*. *pertussis* CyaA (55). Further studies are warranted in order to evaluate the importance of epithelial barrier disruption in the pathogenesis of *B*. *pertussis*.

## MATERIALS AND METHODS

Cultured cell lines, bacterial strains, gene constructions, and other commonly utilized methods are given in Text S1 in the supplemental material.

### Isolation of cytosolic and membrane fractions from CyaA-treated cells.

J774A.1 cells grown to confluence were treated with 0.5 µg/ml of each recombinant CyaA at 37°C for 1 h. After the cells were washed with cold Dulbecco-modified phosphate-buffered saline (D-PBS), the cells were scraped and collected into a tube with prechilled homogenization buffer (25 mM Tris-HCl, 150 mM NaCl, 1 mM EDTA [pH 7.4] containing protease inhibitor cocktail [Nacalai] and a mixture of a phosphatase inhibitor [PhosSTOP; Roche]). The cells were homogenized in a Potter-Elvehjem homogenizer and centrifuged at 1,000 × *g* for 30 min. The supernatant was collected into another tube, and the pellet was resuspended in fresh homogenization buffer and homogenized again. Homogenization and centrifugation were repeated four times, and the supernatant obtained each time was collected into a tube and centrifuged at 100,000 × *g* at 4°C for 30 min. The resulting supernatant was used as the cytosolic fraction. The pellet was resuspended in the homogenization buffer and used as the membrane fraction. Each fraction was validated by immunoblotting with an anti-transferrin receptor monoclonal antibody (Zymed) and anti-GAPDH (glyceraldehyde-3-phosphate dehydrogenase) polyclonal antibody (Santa Cruz).

### Mass analysis of the intracellular 46-kDa fragment of CyaA.

The cytosolic fraction of CyaA-treated J774A.1 cells was immunoprecipitated with the anti-ACD (adenylate cyclase domain) antibody, and the precipitated proteins were subjected to SDS-PAGE, followed by silver staining (silver staining MS kit; Wako) and separately by immunoblotting with the anti-ACD antibody after electrotransfer to polyvinylidene difluoride (PVDF) membranes. The position of ACD in the gel after SDS-PAGE was estimated on the basis of the relative position of ACD detected by immunoblotting, and the piece of the gel including ACD was reduced with 10 mM dithiothreitol (DTT), alkylated with 55 mM iodoacetamide, and digested with trypsin. The resultant peptides were analyzed by nanocapillary reversed-phase liquid chromatography coupled to tandem mass spectrometry (LC-MS/MS) using a C_18_ column on a nanoLC system (Advance; Michrom BioResources) coupled to a linear trap quadropole (LTQ) Orbitrap Velos plus electron transfer dissociation (ETD) mass spectrometer (Thermo Fisher Scientific). Tandem mass spectra were acquired automatically by alternating collision-induced dissociation (CID)/ETD fragmentation and searched against a nonredundant *Bordetella* database from NCBI using the MASCOT Server (Matrix Science). Precursor mass tolerance was set at 10 ppm and 0.8 Da for the Orbitrap and linear ion trap, respectively. Carbamidomethylation (C) was set as the static modification. Oxidized methionine (M), the acetylation of the N terminus, and the phosphorylation of serine, threonine, or tyrosine were set as dynamic modifications. A maximum of two missed cleavage sites were allowed.

### Measurement of transepithelial electrical resistance and immunofluorescence microscopy of CyaA-treated EGV-4T cells.

EGV-4T cells were seeded on a 24-well Transwell plate at 50,000 cells/well and allowed to grow for 5 days in medium, but not in an air-liquid interface until the transepithelial electrical resistance (TEER) reached 2 to 3 kΩ/well. Medium was replaced with fresh medium, and cells were incubated at 37°C for 30 min and then treated with 1 µg/ml of each recombinant CyaA for 24 h. TEER was measured with Millicell-ERS (electrical resistance system) (Merck) every 2 h. After 24 h, the cells were fixed with 4% paraformaldehyde in D-PBS for 15 min, permeabilized with 0.5% Triton X-100 in D-PBS for 5 min, and blocked with 5% bovine serum albumin (BSA) in D-PBS at room temperature for 30 min. The cells were treated with an anti-ZO-1 monoclonal antibody (Invitrogen) for 1 h and subsequently treated with an Alexa Fluor 488-labeled secondary antibody (Molecular Probes) and Hoechst 33258 for 30 min, mounted with the filter in Fluoromount (Diagnostic BioSystems), and subjected to microscopy with a confocal laser scanning microscope (Olympus FluoVIEW FV10i; Olympus). Cell death was detected by the LIVE/DEAD cell imaging kit (Molecular Probes) according to the manufacturer’s instructions.

### Animal experiments.

Three-week-old female Wistar rats (Japan SLC) were anesthetized with a mixture of medetomidine (Kyoritsuseiyaku Co., Tokyo, Japan), midazolam (Teva Takeda Pharma, Nagoya, Japan), and butorphanol (Meiji Seika Pharma Co., Tokyo, Japan) at final doses of 0.3, 2.0, and 5.0 mg/kg of body weight, respectively, and intranasally inoculated with 500 to 700 CFU of B. bronchiseptica in 10 µl of SS medium using a micropipette with a needle-like tip. On day 5 postinoculation, rats were euthanized with pentobarbital, and the tracheas were excised, weighed, and minced by Polytron (Kinematica) in D-PBS for measurements of the number of bacteria that colonized the rat trachea.

Regarding tracheal-epithelial barrier staining, rats were euthanized with pentobarbital, and 1 mg/ml of nonpermeable biotin (sulfo-NHS biotin; Pierce) in 50 µl was injected into the lumen of the trachea. After 3 min, the trachea was flushed out with D-PBS, excised, fixed with 4% paraformaldehyde, embedded in paraffin, and longitudinally sectioned. The sections were immersed in xylene and ethanol for deparaffinization and treated with 10 mM sodium citrate, pH 6.0. After the sections were blocked with D-PBS containing 5% fetal calf serum (FCS) and 0.3% Triton X-100, the sections were stained for biotin, and the nuclei were stained with Alexa Fluor 488 streptavidin (Thermo Fisher) and Hoechst 33258, respectively, and subjected to fluorescence microscopy as described above.

All animal experiments were approved by the Animal Care and Use Committee of the Research Institute for Microbial Diseases, Osaka University, and performed according to the Regulations on Animal Experiments at Osaka University.

10.1128/mBio.00628-18.7 TABLE S1  Primers used for the construction of plasmids. Download TABLE S1, DOCX file, 0.03 MB. Copyright © 2018 Fukui-Miyazaki et al. 2018 Fukui-Miyazaki et al. This content is distributed under the terms of the Creative Commons Attribution 4.0 International license.

10.1128/mBio.00628-18.8 TABLE S2  Primers used for the RT-PCR analysis of 14-3-3. Download TABLE S2, DOCX file, 0.01 MB. Copyright © 2018 Fukui-Miyazaki et al. 2018 Fukui-Miyazaki et al. This content is distributed under the terms of the Creative Commons Attribution 4.0 International license.
