# Supplementary material for: The Eukaryotic Host Factor 14-3-3 Inactivates Adenylate Cyclase Toxins of Bordetella bronchiseptica and B. parapertussis, but Not B. pertussis
Source: mBio. 2018 Aug 28;9(4):e00628-18. doi: 10.1128/mBio.00628-18 (PMC6113625; doi:10.1128/mBio.00628-18)
Supplement: TEXT S1 [file mbo004184038s1.docx]

**Text S1: Supplemental Materials and Methods**

**Cell lines, bacterial strains, and cultures**

L2 (rat type 2 alveolar epithelium), J774A.1 (mouse macrophage), THP-1 (human monocyte), EGV-4T (rat tracheal epithelial), and CHO-K1 (Chinese hamster ovary) cells were grown at 37°C under 5% CO_2_ in air. Ham’s F-12K (Kaighn’s, Gibco) for L2 cells, RPMI 1640 (Gibco) for J774A.1 and THP-1 cells, and Ham’s F-12 (Gibco) for EGV-4T and CHO-K1 cells were used as culture media in the presence of 10% fetal calf serum (FCS, JRH Biosciences).

*Bordetella pertussis* Tohama I and 18323 and *B. bronchiseptica* S798 were maintained in the laboratory. *B. bronchiseptica* RB50 was provided by P.A. Cotter, (University of California, CA, USA). *B. parapertussis* strains 12822 and C277 were provided by A. Abe (Kitasato University, Tokyo, Japan). *B. pertussis*, *B. bronchiseptica*, *B. parapertussis*, and *B. bronchiseptica* ∆*cyaA* transformed with the plasmids encoding CyaA-derived proteins were recovered from colonies on plates of Bordet-Gengou agar (Becton Dickinson) containing 0.4% (w/v) Polypeptone (Wako), 0.8% glycerol, 15-20% defibrinated horse blood, and 10 µg/ml ceftibuten (BG) and then suspended in Stainer and Scholte (SS) medium, followed by an incubation at 37°C for an appropriate period with shaking. Colony-forming units (CFU) were measured by plating serially diluted samples on BG plates and enumerating grown colonies after a two-day cultivation.

*Escherichia coli* was grown on Luria-Bertani (LB) agar or broth. Growth media were supplemented with antibiotics where necessary at the following concentrations: ampicillin, 50 µg/ml; kanamycin, 50 µg/ml; chloramphenicol, 10 µg/ml.

**Construction of expression vectors for CyaA-derived proteins**

The primers used for the construction of plasmids encoding CyaA and its derivatives were listed in Table S1. The plasmids for Bp-Bb and Bb-Bp chimeric CyaAs were generated as follows. The CyaA genes of Bp and Bb were amplified by PCR with the primers HindIII-BP0760-S2 and XbaI-BB0324-AS using *B. pertussis* Tohama I and *B. bronchiseptica* RB50 genomic DNA as the templates, respectively. PCR products were excised with *Hind*III/*Xba*I and inserted into the same sites of the pBBR1MCS5 vector with a broad host range (1). The resulting plasmids were designated as pBBR1MCS5-BP0760 and pBBR1MCS5-BB0324. The ACD-TMD region and RTX region of Bp *cyaA* were amplified by PCR with the primers HindIII-BP0760-S2 and D-XhoI-BB0324-AS, and U-XhoI-BB0324-S and XbaI BB0324-AS, respectively, using *B. pertussis* Tohama I genomic DNA as a template. The resulting PCR products were cloned into pCR™4Blunt-TOPO (Zero Blunt TOPO PCR Cloning Kit, Invitrogen), sequenced, and digested with *Hind*III and *Xho*I or *Xba*I and *Xho*I, respectively. The digested fragments were inserted into the same sites of pBBR1MCS5-BB0324 and the plasmids obtained were designated pBBR1MCS5-BP/BB and pBBR1MCS5-BB/BP, respectively. The plasmids for Bp-Bb chimeric CyaAs with a point mutation were generated by the site-directed mutagenesis method using the primers described in Table S1 and pBBR1MCS5-BP/BB as the template.

Regarding the production of the Bp CyaA mutants, F375S and V800A, pBBR1MCS5-BP/BB carrying the corresponding amino-acid replacements were digested with *Hind*III and *Xho*I, and the digested fragments were independently inserted into the same sites of pBBR1MCS5-BP0760. The resulting plasmids were named pBBR1MCS5-BP0760-F375S and pBBR1MCS5-BP0760-V800A, respectively. In the production of the Bb CyaA mutants, S375F and A800V, the gene for the N-terminal region of Bb CyaA in pCR™4Blunt-TOPO (see supporting information) was amplified by PCR with the primer sets BB>BP-S375F-S/BB>BP-S375F-AS and BB>BP-A800V-S/BB>BP-A800V-AS, followed by digestion with *Hind*III and *Xho*I. The digested fragments were inserted into the same sites of pBBR1MCS5-BB0324 and the plasmids obtained were designated pBBR1MCS5-BB0324-S375F and pBBR1MCS5-BB0324-A800V, respectively. These constructed plasmids were introduced into *B. bronchiseptica ∆cyaA* by electroporation.

Bp CyaA and an enzyme-dead mutant of CyaA (K58Q) (2) were produced by *E. coli* JM109 harboring pCACT3 (3) and pCACT3-K58Q, respectively. In order to obtain *E. coli* producing the recombinant CyaA proteins, we generated expression plasmids as described below. Site-directed mutagenesis was performed to replace Phe^375^ of Bp CyaA with Ser^375^ using the primers BP>BB-3-S and BP>BB-3-AS with pCACT3 as the template. Regarding Bb CyaA, pCACT3-BB was constructed by exchanging the Bp cyaA gene in pCACT3 for the Bb cyaA gene, and the codon for Ser^375^ in this construct was replaced with that for Phe by site-directed mutagenesis using BB>BP-S375F-S and BB>BP-S375F-AS. Recombinant CyaAs were produced by *E. coli* JM109 harboring each expression vector described above in the presence of 1 mM IPTG. The induced insoluble products from bacterial cells were solved in 50 mM Tris-HCl, pH 8.0, containing 8 M urea and 0.2 mM CaCl_2_, followed by DEAE-Sepharose (GE Healthcare) chromatography. The fractions containing the toxins were eluted with a linear gradient of NaCl from 0.1 M to 0.5 M in the same buffer.

The mutant strain of *B. bronchiseptica* producing Bb CyaA_S375F_ (Bb S375F) was established as follows. The full-length DNA of Bb CyaA with a mutation at the 375^th^ amino-acid position was amplified by PCR with the primers BB0324-1660-F and BB0324-2755-R using pBBR1MCS5-BB0324-S375F as the temple. The PCR product was cloned into pCR™4Blunt4-TOPO, sequenced, and digested with *Not*I and *Spe*I. The digested fragment was inserted into the same sites of pABB-CRS2 (4), which was gifted by A. Abe. The resulting plasmid was introduced into *E. coli* SM10 λ*pir* and transconjugated into *B. bronchiseptica* RB50 by biparental conjugation. Similarly, the mutant strain of *B. pertussis* producing Bp CyaA_F375S_ (Bp F375S) was generated using pBBR1MCS5-BP0760-F375S as the template for PCR. The resulting plasmid was introduced into *B. pertussis* Tohama I. Genes constructed and introduced by conjugation as described above were integrated into the corresponding regions of parental strains by two-step homologous recombination. The integration of the introduced genes was confirmed by sequencing.

*B. bronchiseptica* ∆*cyaA* and *B. pertussis* ∆*cyaA* were generated by a previously described method (5) using the primers shown in Table S1 and genomic DNA from *B. bronchiseptica* RB50 and *B. pertussis* Tohama I as the templates.

Genes coding the 2^nd^~400^th^ amino-acid region of Bp CyaA (Bp ACD) or Bp CyaA_F375S_ (Bp ACD_F375S_) with the N-terminal FLAG and C-terminal 6x His peptide tags were amplified by PCR with the primers FLAG-ACT F1 and ACT-His stop R1, and pBBR1 MCS5-BP/BB for Bp ACD and pBBR1 MCS5-BpF375S for Bp ACD_F375S_ as the templates. The amplified fragments were cloned into pCR™4Blunt4-TOPO, sequenced, excised with *EcoR*I, and inserted into the same site of pET21a. Bp ACD and Bp ACD_F375S_ were produced in *E. coli* BL21-CodonPlus (DE3) RIL incubated at 37°C for 3 h with 1 mM of IPTG. The insoluble products induced from bacterial cells, which contained either ACD mutant, were solved in 50 mM Tris-HCl (pH 8.0) containing 150 mM NaCl and 8 M urea.

The cyaA gene with a mutation in the codon for Val (-34) or Met (+1) was constructed as follows. An amount of the *cyaA* gene encoding the N-terminal region of Bb CyaA was amplified by PCR with the primers HindIII-BP0760-S2 and D-XhoI-BB0324-AS using *B. bronchiseptica* RB50 genomic DNA as the template, cloned into pCR™4Blunt-TOPO, and sequenced. The replacement of the codon GTG for Val (-34) with GGG for Gly or the codon ATG for Met (+1) with ATC for Ile was generated by the site-directed mutagenesis method using the primers GTG>GGG-S and GTG>GGG-AS or ATG>ATC-S and ATG>ATC-AS, respectively, and the plasmid described above as the template. DNA fragments with the mutations were excised with *Hind*III and *Xho*I and inserted into the same sites of pBBR1MCS5-BB0324.

**Production of 14-3-3 proteins**

The mammalian expression plasmids for each isoform of human 14-3-3 proteins were kindly gifted by M. Inoue (Kurume Univ., Fukuoka, Japan), and A. Tsuji and K. Yuasa (Tokushima Univ., Tokushima, Japan) (6). In order to produce the 14-3-3 proteins in *E. coli*, a *Nde*I site was inserted upstream of the gene for the FLAG-tag in each plasmid for the 14-3-3 isotype by site-directed mutagenesis, and the genes encoding the FLAG-tag and each isoform of the 14-3-3 protein were excised from the plasmids by digestion with *Nde*I and *Sal*I and then inserted into the *Nde*I-*Sal*I sites of pET28b. Each plasmid was introduced into *E. coli* BL21 (DE3) and 14-3-3 proteins were produced by the bacteria incubated at 20°C overnight in the presence of 1 mM of IPTG. Bacterial cells were sonicated in cold 50 mM Tris-HCl, pH 7.2, and the supernatants obtained after centrifugation were used as FLAG-14-3-3-containing *E. coli* fractions.

**Antibodies against CyaA**

Rabbit anti-CyaA antiserum was generated by Keari Co. (Japan) using purified recombinant Bp CyaA as an antigen (7). The IgG fraction was purified from antiserum by protein G- Sepharose (GE Healthcare) chromatography and used as an anti-CyaA polyclonal antibody. The anti-CyaA monoclonal antibodies 3D1 and 9D4 were purchased from Santa Cruz Biotechnology. An anti-ACD polyclonal antibody was purified from anti-ACT antiserum by the affinity chromatography of Bp ACD-coupled Affi-Gel 15 (Bio-Rad) according to the manufacturer’s instructions.

**Cell rounding assay**

L2 cells were seeded on a 24-well plate at 20,000 cells/well and grown overnight. Medium was replaced with the culture supernatant of *Bordetella* or purified CyaA diluted with Ham’s F-12K containing 1% FCS, which had been maintained at 37°C. After a 3~6-h incubation at 37°C, cells were examined for morphological changes under a phase-contrast microscope. The culture supernatant for the cell rounding assay was prepared as follows. An overnight culture of test bacteria was diluted to an OD_650_ value of 0.02 with Ham’s F-12K medium supplemented with 1% FCS, and incubated at 37°C for 6 h in 5% CO_2_ without shaking. The resulting culture was diluted with the medium to give a concentration of 6.6 × 10^7^ bacterial cells/ml (OD650 = 0.02), centrifuged at 10, 000 × *g* for 30 min, and subjected to the cell rounding assay after filtration through a 0.22-µm pore PVDF membrane (Millipore). In SDS-PAGE and immunoblotting, an aliquot of the culture supernatant was concentrated 25-fold by trichloroacetic acid precipitation and 10 µl of each concentrated sample was applied for electrophoresis.

**Adenylate cyclase assay**

The adenylate cyclase activities of CyaA and its derivatives were assessed as follows. Toxin preparations were incubated with 2 mM of ATP in reaction buffer (60 mM Tris-HCl, pH 8.0, containing 7 mM MgCl_2_, 0.1 mM CaCl_2_, 1 mg/ml of BSA, and 100 nM calmodulin) at 37°C for 20 min. The reaction was stopped by the addition of a 2-fold volume of 0.5 M HCl and boiled for 5 min. After the addition of the same volume of 1.5 M imidazole to neutralize the pH of the solution, the amount of cAMP was measured with the cAMP Biotrak enzyme immunoassay system (cAMP EIA system, GE Healthcare) according to the manufacturer’s instructions.

The amount of intracellular cAMP in cells treated with CyaAs was assessed as follows. Cells in a 96-well plate were cultured overnight, and then incubated with each concentration of the recombinant toxin diluted with HBSS, 20 mM Hepes-NaOH, pH 7.4, containing 0.1% BSA (Hank’s-Hepes-BSA) for 1 h. Cells were lysed with the lysis buffer provided with the cAMP EIA system and the amount of cAMP was measured as described above.

***In vitro* infection assay**

Wild-type *B. pertussis* Tohama I and *B. bronchiseptica* RB50 were recovered from colonies on BG plates and cultured in SS medium at 37°C for 25 and 7 h, respectively, until the OD_650_ value was 1~2. The bacteria that grew were washed and resuspended in Hank’s-Hepes BSA at a concentration of 2.5 or 1.0 × 10^7^ cfu/ml, and 100 µl of each suspension was added to the wells of a 96-well plate, in which L2 or J774A.1 cells was inoculated at 2.5 or 5.0 × 10^4^ cells/well, respectively, and cultured overnight. After centrifugation of the plate at 500 × *g* for 10 min, cells were incubated with the bacteria at 37°C for 1 h. Cells were lysed with lysis buffer and the amount of cAMP was measured as described in “Adenylate cyclase assay”. The mutant strains producing CyaA with the 375^th^ amino-acid replacement (Bp F375S and Bb S375S) and the *cyaA*-deficient strain (*∆cyaA*) originating in each *Bordetella* were prepared in a similar manner to the wild type.

**Cell binding assay**

L2 cells were incubated with 5 µg/ml of each recombinant CyaA diluted with Hank’s-Hepes-BSA on ice for 30 min, sequentially with an anti-CyaA polyclonal antibody for 1 h, and with AlexaFlour®488-labeled goat anti-rabbit IgG (Molecular Probes) for 30 min. Cells were analyzed using a FACSCalibur flow cytometer (BD Biosciences).

**Hemolysis assay**

The hemolytic activity of CyaA was examined as previously described (8). Briefly, sheep erythrocytes suspended in TNC buffer (10 mM Tris-HCl, pH 7.4, containing 150 mM NaCl, and 1 mM CaCl_2_) were placed at 1 × 10^8^ cells/well in a round-bottomed 96-well plate, and incubated with recombinant CyaAs at 37°C for 5 h. Samples were centrifuged to remove unlysed erythrocytes, and the A_541_ of the supernatant was measured. The level of hemolysis was expressed as the percentage of hemolysis caused by 0.5% Triton X-100.

**Translocation assay**

A translocation assay was performed as previously described (9) with a slight modification. Sheep erythrocytes (1 × 10^9^ cells/ml) were washed with TNC buffer and incubated with 10 ng/ml of recombinant CyaAs at 37 or 4°C for appropriate periods up to 120 min. The reaction was stopped by the addition of and an incubation with the same volume of TNE buffer (10 mM Tris-HCl, pH 7.4, containing 150 mM NaCl and 2 mM EDTA) on ice for 5 min. Cells were treated with 50 µg/ml of TPCK-trypsin at 37°C for 15 min, followed by the addition of a trypsin inhibitor at a final concentration of 100 µg/ml. After washing with TNE buffer, cells were collected by centrifugation, lysed with the lysis buffer of the cAMP EIA system, and subjected to cAMP measurements.

**RT-PCR analysis**

The total RNA of J774A.1 cells was obtained by TRIZOL reagent (Thermo Fisher Scientific) according to the manufacturer’s instructions. RT-PCR was performed with primers specific for each 14-3-3 isoform described in Table S2 under the following conditions: initial denaturation at 94°C for 1 min followed by 30 cycles of denaturation at 98°C for 10 sec, annealing at 60°C for 30 sec, and an extension at 72°C for 30 sec. PCR products were separated on a 1% (w/v) agarose gel.

**Others**

The CyaA preparations used in the present study were appropriately diluted with 50 mM Tris-HCl, pH 8.0, containing 0.2 mM CaCl_2_ and 8 M urea, and each dilution was further diluted 100-fold just before the assays such that urea was present at equal concentrations in each test group.

The protein concentrations of supernatants were measured using BCA Protein Assay Reagents (Thermo Scientific) according to the manufacturer’s instructions.

Immunoblotting was performed as follows. After SDS-PAGE, proteins in gels were transferred to PVDF membranes (Millipore), which were blocked with 10% skimmed milk and subsequently incubated with an antibody against each target protein, followed by peroxidase-conjugated goat anti-rabbit IgG (Jackson ImmunoResearch) or peroxidase-conjugated goat anti-mouse IgG (Cappel). The target proteins were detected by a LAS-4000 luminescence image analyzer using an enhanced chemiluminescence system (TaKaRa) in accordance with the manufacturer’s instructions. The density of the bands was quantified by a densitometric analysis using Image Gauge Version 4.1 (Fujifilm, Tokyo, Japan).

Regarding immunoprecipitation, the anti-ACD antibody was coupled to Pierce NHS-Activated Magnetic Beads (Thermo Scientific) according to the manufacturer’s instructions. Samples were incubated with antibody-linked beads at room temperature for 2 h with rotation. After washing with IP Lysis/Wash Buffer (Thermo Scientific), the beads were boiled with 1X dye (Thermo Scientific) for 5 min, and the eluate containing bound proteins was collected.

Bp ACD or Bp ACD_F375S_ was phosphorylated by an incubation with or without PKA at a molar ratio of 8:1 at 37°C for 20 min in 50 mM Tris-HCl, pH 7.2, containing 10 mM MgCl_2_, 5 mM DTT, 200 µM ATP, and the mixture of a phosphatase inhibitor (PhosSTOP).

In order to concentrate proteins for the analysis by SDS-PAGE, samples were mixed with the same volume of 20% trichloroacetic acid (TCA) and allowed to stand on ice for 20 min. The resulting precipitate was sequentially washed with cold 100% ethanol and suspended in the loading dye.

**References related to supplemental material.**

1. **Kovach ME**, **Elzer PH**, **Hill DS**, **Robertson GT**, **Farris MA**, **Roop RM**, **Peterson KM**. 1995. Four new derivatives of the broad-host-range cloning vector pBBR1MCS, carrying different antibiotic-resistance cassettes. Gene **166**:175–176.

2. **Glaser P**, **Elmaoglou-Lazaridou A**, **Krin E**, **Ladant D**, **Bârzu O**, **Danchin A**. 1989. Identification of residues essential for catalysis and binding of calmodulin in *Bordetella pertussis* adenylate cyclase by site-directed mutagenesis. EMBO J **8**:967–972.

3. **Betsou F**, **Sebo P**, **Guiso N**. 1993. CyaC-mediated activation is important not only for toxic but also for protective activities of *Bordetella pertussis* adenylate cyclase-hemolysin. Infect Immun **61**:3583–3589.

4. **Sekiya K**, **Ohishi M**, **Ogino T**, **Tamano K**, **Sasakawa C**, **Abe A**. 2001. Supermolecular structure of the enteropathogenic *Escherichia coli* type III secretion system and its direct interaction with the EspA-sheath-like structure. Proc Natl Acad Sci USA **98**:11638–11643.

5. **Nishikawa S**, **Shinzawa N**, **Nakamura K**, **Ishigaki K**, **Abe H**, **Horiguchi Y**. 2016. The bvg-repressed gene brtA, encoding biofilm-associated surface adhesin, is expressed during host infection by *Bordetella bronchiseptica*. Microbiology and Immunology **60**:93–105.

6. **Yuasa K**, **Ota R**, **Matsuda S**, **Isshiki K**, **Inoue M**, **Tsuji A**. 2015. Suppression of death-associated protein kinase 2 by interaction with 14-3-3 proteins. Biochem Biophys Res Commun **464**:70–75.

7. **Abe H**, **Kamitani S**, **Fukui-Miyazaki A**, **Shinzawa N**, **Nakamura K**, **Horiguchi Y**. 2015. Detection of genes expressed in *Bordetella bronchiseptica* colonizing rat trachea by in vivo expressed-tag immunoprecipitation method. Microbiology and Immunology **59**:249–261.

8. **Bellalou J**, **Sakamoto H**, **Ladant D**, **Geoffroy C**, **Ullmann A**. 1990. Deletions affecting hemolytic and toxin activities of *Bordetella pertussis* adenylate cyclase. Infect Immun **58**:3242–3247.

9. **Rogel A**, **Hanski E**. 1992. Distinct steps in the penetration of adenylate cyclase toxin of *Bordetella pertussis* into sheep erythrocytes. Translocation of the toxin across the membrane. J Biol Chem **267**:22599–22605.

10. **Park J**, **Zhang Y**, **Buboltz AM**, **Zhang X**, **Schuster SC**, **Ahuja U**, **Liu M**, **Miller JF**, **Sebaihia M**, **Bentley SD**, **Parkhill J**, **Harvill ET**. 2012. Comparative genomics of the classical *Bordetella* subspecies: the evolution and exchange of virulence-associated diversity amongst closely related pathogens. BMC Genomics **13**:545.
