# Supplementary material for: The Eukaryotic Host Factor 14-3-3 Inactivates Adenylate Cyclase Toxins of Bordetella bronchiseptica and B. parapertussis, but Not B. pertussis
Source: mBio. 2018 Aug 28;9(4):e00628-18. doi: 10.1128/mBio.00628-18 (PMC6113625; doi:10.1128/mBio.00628-18)
Supplement: TABLE S1 [file mbo004184038st1.docx]

**Table S1. Primers used for the construction of plasmids**

| Name | Sequence | Application |
| --- | --- | --- |
| HindIII-BP0760-S2  D-XhoI-BB0324-AS  U-XhoI-BB0324-S  XbaI-BB0324-AS | CGGAAGCTTTTGCTCGCTTATTTATCTCC  CGCATTGCCGGTGATCGAAT  GCGCACGGAGAATGTCCAATACCG  CCCTCTAGAATGACCAGGCAGAGCAACCCGGAAT | Chimeric CyaAs |
| BP>BB-2-S  BP>BB-2-AS | GTGCCGGGCGGACGTTCGAAG  CTTCGAACGTCCGCCCGGCAC | Bp-Bb chimeric CyaA S370G (AGC→GGC) |
| BP>BB-3-S  BP>BB-3-AS | GACGTTCGAAGTCCTCGCCGGATG  CATCCGGCGAGGACTTCGAACGTC | Bp-Bb chimeric CyaA  F375S (TTC→TCC) |
| BP>BB-5-S  BP>BB-5-AS | CCTGAAATCCGCCGACGTGTTCG  CGAACACGTCGGCGGATTTCAGG | Bp-Bb chimeric CyaA  V800A (GTC→GCC) |
| BP>BB-6-S  BP>BB-6-AS | GGACCGCTTCATCCAGGGCGAG  CTCGCCCTGGATGAAGCGGTCC | Bp-Bb chimeric CyaA  V808I (GTC→ATC) |
| BP>BB-7-S  BP>BB-7-AS | GCATCAAACTGGAGGTGATCGG  CCGATCACCTCCAGTTTGATGC | Bp-Bb chimeric CyaA  D910E (GAT→GAG) |
| BP>BB-8-S  BP>BB-8-AS | GGCTACCCAGAAAACCGCCTACG  CGTAGGCGGTTTTCTGGGTAGCC | Bp-Bb chimeric CyaA  T978K (ACA→AAA) |
| BB>BP-S375F-S  BB>BP-S375F-AS | GACGTTCGAAGTTCTCGCCGGATG  CATCCGGCGAGAACTTCGAACGTC | Bb CyaA mutant  S375F (TCC→TTC) |
| BB>BP-A800V-S  BB>BP-A800V-AS | CCTGAAATCCGTCGACGTGTTCG  CGAACACGTCGACGGATTTCAGG | Bb CyaA mutant  A800V (GGC→GTC) |
| GTG>GGG-S  GTG>GGG-AS | GTCATACGACGGGCTGGATGTTTGG  CCAAACATCCAGCCCGTCGTATGAC | Val (-34) mutation |
| ATG>ATC-S  ATG>ATC-AS | CAGATCCACATCCAGCAATCGC  GCGATTGCTGGATGTGGATCTG | Met (1) mutation |
| BB0324-1660-F  BB0324-2755R | GCGGATATCGACATGTTC  TCATCGGTCAACGACATC | Bb producing CyaA_S375F_  Bp producing CyaA_F375S_ |
| RB50-ACT-U-F  RB50-ACT-U-R  RB50-ACT-D-F  RB50-ACT-D-R | GATCCGAGCTCTCCCTGGCCGAGTACATGCAGA  TTTAGCTTCCTTAGCTCTGATGCGATTGCTGCATGT  CGCGCCATTTAAATGGCTGTCAACTGGCGCTGA  ATTTGTGGAATTCCCGAAAATAGCAAGGCAGCG | Bb ∆*cyaA* |
| BP0760 U-S+  BP0760 U-AS+  BP0760 D-S+  BP0760 D-AS+ | GATCCGAGCTCTCCCGTTGCTCGCGCGCAGTC  TTAGCTTCCTTAGCTCTGATGCGATTGCTGCATGT  CGCGCCATTTAAATGGCTGTCAACTGGCGCTGA  ATTTGTGGAATTCCCAAATAGCAAGGCAGCGCCG | Bp *∆cyaA* |
| FLAG-ACT-F1  ACT-His stop-R1 | ATGGATTACAAGGATGACGACGATAAGCAGCAATCG  CATCAGGCTGGTTACG  TCAGTGATGGTGATGGTGATGCTGGCGTTCCACTGC  GCCCAGCGAC | ACDs |
