# Supplementary material for: The Eukaryotic Host Factor 14-3-3 Inactivates Adenylate Cyclase Toxins of Bordetella bronchiseptica and B. parapertussis, but Not B. pertussis
Source: mBio. 2018 Aug 28;9(4):e00628-18. doi: 10.1128/mBio.00628-18 (PMC6113625; doi:10.1128/mBio.00628-18)
Supplement: TABLE S2 [file mbo004184038st2.docx]

**Table S2. Primers used for the RT-PCR analysis of 14-3-3**

| 14-3-3 isotype Sequence Source |
| --- |
| Beta F GAACGTGGTAGGTGCCCGCC Reference*  R GGCCAGGCTGCAGGCCTTTT |
| Epsilon F GACCGTGCCTGCAGGTTGGC Reference*  R CTTGCCAGTGTGGCCGGAGA |
| Eta F GATATGGCCTCCGCCATGAAGGCG Reference*  R CATCCTGCTGGTCGCTCGTCCAGAG |
| Gamma F TCCCTCCGACACACGAGCTCCAA Reference*  R TGGCCACTTCTGCCAGGTAACG |
| Sigma F CAGTTCGCCCGTCTGTCTGTCCA Reference*  R GATGGGGTTGGTAGGCGGCATC |
| Theta F AGCCAATGCAACTAATCCAG This study  R GTTTGTTTTCGATCATCACCA |
| Zeta F GTGTCTGCGGAGCGGCTGTAGC Reference*  R TCTGGTTGCGAAGCATTGGGGA |
| β-actin F GGGAAATCGTGCGTGACATT This study  R GCGGCAGTGGCCATCTC |

***Reference**: J. Meng, C. Cui, Y. Liu, M. Jin, D. Wu, C. Liu, E. Wang, and B. Yu, PLoS ONE 8:e53633, 2013, doi:10.1371/journal.pone.0053633
